# Nano-bio interactions: a neutrophil-centric view

**DOI:** 10.1038/s41419-019-1806-8

**Published:** 2019-07-29

**Authors:** Sandeep Keshavan, Paolo Calligari, Lorenzo Stella, Laura Fusco, Lucia Gemma Delogu, Bengt Fadeel

**Affiliations:** 1 Institute of Environmental Medicine, Karolinska Institutet, Stockholm, Sweden; 2 Department of Chemical Sciences and Technologies, University of Rome Tor Vergata, Rome, Italy; 3 Department of Chemical and Pharmaceutical Sciences, University of Trieste, Trieste, Italy; 4 Department of Biomedical Sciences, University of Padua, Padua, Italy; 5 Fondazione Istituto di Ricerca Pediatrica Città della Speranza, Padua, Italy

**Keywords:** Cell signalling, Mechanisms of disease

## Abstract

Neutrophils are key components of the innate arm of the immune system and represent the frontline of host defense against intruding pathogens. However, neutrophils can also cause damage to the host. Nanomaterials are being developed for a multitude of different purposes and these minute materials may find their way into the body through deliberate or inadvertent exposure; understanding nanomaterial interactions with the immune system is therefore of critical importance. However, whereas numerous studies have focused on macrophages, less attention is devoted to nanomaterial interactions with neutrophils, the most abundant leukocytes in the blood. We discuss the impact of engineered nanomaterials on neutrophils and how neutrophils, in turn, may digest certain carbon-based materials such as carbon nanotubes and graphene oxide. We also discuss the role of the corona of proteins adsorbed onto the surface of nanomaterials and whether nanomaterials are sensed as pathogens by cells of the immune system.

## Known facts

- Nanomaterials are inevitably cloaked with proteins giving rise to a bio-corona.
- Nanomaterials can trigger inflammation with activation of the inflammasome.
- Carbon-based materials may undergo digestion by macrophages or neutrophils.

## Open questions

- Does the innate immune system sense engineered nanomaterials as pathogens?
- Are nanomaterial-induced neutrophil extracellular traps or NETs good or bad?
- Can nanomaterials elicit exosome-mediated pro- or anti-inflammatory signals?

## Introduction

Inflammation is a complex biological response involving soluble factors and cells that arises in a tissue in response to harmful stimuli including pathogens, toxicants, or dead cells. The process normally leads to recovery and healing. However, inflammation can also lead to persistent tissue damage and may even promote neoplastic transformation^1,2^. Indeed, the distinction between acute and chronic inflammation is important, not least in toxicology. Inflammation is fundamentally a normal, protective physiological response to injury or infection. However, if inflammatory responses are persistent due to an exaggerated or dysregulated response, including failure of resolution of inflammation, a pathological response occurs^3^.

Understanding interactions of engineered nanomaterials with the immune system is of considerable relevance both from a toxicological and biomedical perspective^4^. However, whereas numerous publications have focused on nanomaterial interactions with macrophages, less attention is devoted to neutrophils, despite the fact that neutrophils are key factors in inflammation. In fact, research in recent years has revealed that these cells may also inform and shape adaptive immune response, in addition to their traditional roles as hunters and killers of microbes^5^. Furthermore, although this has previously been overlooked, neutrophils also express a rich repertoire of so-called pattern recognition receptors or PRRs^6^. We will focus here on the interactions of engineered nanomaterials with neutrophils, the most abundant of the white blood cells.

## Nanomaterial effects on neutrophils

Neutrophils are key factors in inflammation and numerous studies have shown that engineered nanomaterials may elicit acute and/or chronic inflammation in different animal models^7^. However, despite numerous studies showing tissue infiltration of neutrophils upon exposure to nanoparticles, it can be argued that neutrophils are a somewhat neglected cell in nanotoxicology, as there are relatively few studies on direct interactions of nanomaterials with these cells. Nevertheless, neutrophils are normally the first responders in an inflammatory reaction while macrophages arrive in the second wave of inflammation and serve mainly to remove cell debris and to promote tissue healing^8^. Similarly, it is worth noting that macrophages are not the only cells that are involved in the clearance of nanoparticles from the blood; in fact, a recent study showed that neutrophils also play a major role in nanoparticle clearance, at least in some mouse strains^9^. Notably, although neutrophils are cleared from the circulation via the liver and spleen, evidence has been put forward that the bone marrow is a major site of neutrophil clearance^10^. It follows that nanoparticles that are cleared from the circulation by neutrophils could end up in the bone marrow and yet the bone marrow is frequently overlooked as a possible site for the sequestration of nanoparticles, as particle uptake by macrophages in the liver or spleen is usually in focus^11^.

Girard and colleagues^12–15^ have published a series of papers in which various metal and metal oxide nanoparticles including nanoparticles of titanium dioxide, zinc oxide, and silver were shown to activate neutrophils and/or to inhibit neutrophil apoptosis. In contrast, gold nanoparticles were found to activate or accelerate neutrophil apoptosis^16^. Needless to say, careful attention to endotoxin contamination of the tested particles is required^17^. Other authors have shown that silver nanoparticles differently affect distinct subpopulations of neutrophils^18^. Fromen et al.^19^ documented interactions between injected nanoparticles and circulating neutrophils, which could drive particle clearance but could also alter neutrophil responses in a mouse model of acute lung injury. The attachment of poly(ethylene glycol) (PEG) onto the surface of nanoparticles is commonly thought to prevent particle opsonization and macrophage uptake. However, a recent study suggested, instead, that neutrophils preferentially internalized PEGylated particles (i.e., polystyrene microspheres) in the presence of human plasma^20^. Notably, when the authors used model cell lines such as HL-60 or THP-1 cultured in standard cell medium supplemented with 10% fetal bovine serum, PEGylation reduced uptake of the particles. However, when these cells were cultured in human plasma, the PEGylated particles were more avidly taken up, in line with the results obtained with primary cells^20^. This suggests that nanomedicine approaches based on PEGylation of nanoparticles need to be reconsidered—perhaps the administered particles with their “shield” of surface-attached polymers are being sequestered by neutrophils in the blood? Bisso et al.^21^ conducted an in-depth study of nanomaterial interactions with human neutrophils focusing on polymeric and liposomal particles ranging in size from 20 nm to 5 µm. The authors found that nanoparticles were readily internalized by neutrophils ex vivo in the absence of serum proteins, and that the internalization was size-dependent insofar as a significant increase in uptake of the 200 nm particles was observed over particles < 100 nm in diameter. The inclusion of albumin in the cell culture medium prevented uptake of polystyrene particles and reduced the uptake of liposomal nanoparticles, but enhanced neutrophil uptake of poly(lactic-*co*-glycolic acid) (PLGA) particles. Notably, particle-laden neutrophils (i.e., 1 µg/mL of polystyrene particles or 5 µg/mL of liposomes) were found to undergo normal degranulation upon stimulation with conventional agonists^21^.

Carbon-based nanomaterials, including carbon nanotubes (CNTs), are widely studied in nanotoxicology^22^ and these materials were shown to trigger apoptosis and/or autophagic cell death in macrophages (Box 1). Less is known in regards to neutrophils. In a recent study conducted in the frame of the Horizon2020 project BIORIMA, the toxicity of three multi-walled CNTs (MWCNTs) with varying physicochemical properties was evaluated in neutrophils vs. macrophages. Macrophages were susceptible only to the fiber-like MWCNTs, but neutrophil cell viability was significantly affected by all three CNTs, both long and tangled (Keshavan et al., manuscript in preparation). Thus, although macrophages are capable of ingesting nanomaterials and are widely used as a model in nanotoxicology, neutrophils should not be ignored.

Box 1 Cell death: implications for nanotoxicology The Nomenclature Committee on Cell Death recently provided guidelines for molecular definitions of various cell death modalities^128^, although this is hardly the first nor the last attempt at defining cell death^129^. It is important to bear in mind that nanomaterials can elicit different modes of cell death depending on the material as well as on the cell type, and that the mitigation of the adverse effects of such materials requires the correct diagnosis of cell death^130^.

*Apoptosis*: Regulated cell death precipitated by caspases, either through plasma membrane receptor ligation (extrinsic apoptosis) or via perturbation of mitochondria (intrinsic apoptosis). Nanomaterials such as CNTs trigger apoptosis, for instance in lung cells^131^, but chronic, low-dose exposure may result in apoptosis resistance and oncogenic transformation^132^.

*Pyroptosis*: Regulated cell death associated with the formation of pores in the plasma membrane by gasdermin proteins, usually provoked by caspase-1 activation^133^. Rare earth metal nanoparticles were found to trigger pyroptosis in macrophages and apoptosis in hepatocytes^134^.

*Necroptosis*: Regulated cell death that transpires with RIP1/RIP3 activation and subsequent plasma membrane permeabilization by MLKL (mixed-lineage kinase domain-like pseudokinase)^135^. Few, if any, studies have reported evidence of nanoparticle-induced necroptosis.

*Ferroptosis*: Novel, iron-dependent cell death characterized by lipid peroxidation that is subject to regulation by GPX4 (glutathione peroxidase 4) and tightly linked to glutathione synthesis^136^. Importantly, a recent study revealed that nanoparticles trigger different forms of cell death (i.e., apoptosis or ferroptosis) depending on subtle variations in nanoparticle surface properties^137^.

*Autophagic cell death*: Cell death that requires components of the autophagic machinery (note that autophagic cell death should not be confused with autophagy, a cell survival mechanism). Nanoparticles with a high propensity to release toxic ions may harness autophagy to trigger cell death^138^.

## Bio-corona formation on nanoparticles

Nanomaterials promptly adsorb biomolecules leading to the formation of a so-called bio-corona^23^. The binding of proteins or other biomolecules to nanoparticle surfaces may thereby afford a new “identity” to the nanoparticle^24^. Deng et al.^25^ showed that poly(acrylic acid)-coated gold nanoparticles bind fibrinogen, a protein involved in blood clot formation, in a charge-dependent manner, inducing unfolding of the protein, and that binding to integrin receptors on the surface of the monocytic cell line, THP-1 leads to activation of the nuclear factor-κB pathway and secretion of the pro-inflammatory cytokine tumor necrosis factor-α. In a comprehensive study using a panel of silica and polystyrene nanoparticles of various sizes and surface modifications, Tenzer et al.^26^ could show that plasma protein adsorption occurs very rapidly, and that it affects hemolysis, thrombocyte activation, cellular uptake, and endothelial cell death. Vlasova et al.^27^ reported that adsorbed plasma proteins influenced neutrophil responses caused by polymer-coated, single-walled CNTs (SWCNTs). Specifically, the adsorption of IgG resulted in neutrophil activation, as determined by degranulation and release of myeloperoxidase (MPO). Similarly, protein adsorption modulated neutrophil responses toward carboxylated, non-PEGylated SWCNTs^28^. Lara et al.^29^ employed an immuno-mapping technique to study epitope presentation of two major proteins in the serum corona, low-density lipoprotein (LDL) and immunoglobulin G. The authors could show that both proteins displayed functional motifs allowing for recognition of the bio-corona on silica nanoparticles by LDL receptor and Fc-γ receptor I, respectively. On the other hand, others have pointed out that the bio-corona could shield targeting ligands on nanoparticles^30^. However, recent studies suggested clever ways in which to circumvent this problem^31,32^. Furthermore, purposeful surface modification of CdSe/ZnS quantum dots to induce a protein misfolding event in the bio-corona enabled receptor-mediated endocytosis of the particles^33^. Bisso et al.^21^ reported that the presence of serum reduced the ex vivo uptake of poly(styrene) nanoparticles and liposomes by neutrophils and enhanced the uptake of micro- and nanosized PLGA particles. However, the composition of the bio-corona and the role of specific proteins for neutrophil uptake, or lack thereof, was not examined. Furthermore, neutrophils were found to preferentially internalize PEGylated particles^20^. The authors noted that this is linked to factor(s) in human plasma and provided some evidence for a role of complement. Complement factors also facilitate phagocytosis of apoptotic cells and microbes^4^.

Viruses are natural nanoparticles and it is not surprising that a bio-corona of proteins may form on viruses in various biological fluids, or that the bio-corona may affect immune responses to viruses^34^. Indeed, the adsorbed bio-corona could be considered as part of the motifs that are sensed by immune cells. Hence, the boundaries between pathogen-associated molecular patterns (PAMPs) and damage-associated molecular patterns (DAMPs) apparently begin to dissolve at the nano-bio interface, as both engineered and natural nanoparticles (i.e., viruses) adsorb host proteins on their surface. This topic is discussed in more detail below.

## Exosomes: message in a bottle

Exosomes are nanosized extracellular vesicles that are naturally secreted by cells and they may play a particularly important role in conveying information between immune cells^35^. Exosomes were initially thought to fulfill a janitorial function by providing the cell with a means of getting rid of non-functional proteins and other molecules. Subsequent studies suggested important roles of exosomes in intercellular communication and the interest in exosomes and other microvesicles has escalated in the past two decades due to their emerging roles in health and disease^36^. Exosomes thus harbor specific proteins and nucleic acids, mostly small RNAs, such as ribosomal RNA, transfer RNA, microRNA, and mRNA molecules^36^. Exosomes from activated neutrophils were recently reported to acquire surface-bound neutrophil elastase (NE) and the exosomes were shown to degrade extracellular matrix components causing the hallmarks of chronic obstructive pulmonary disease^37^. Exosomal NE was much more potent than free NE. In the latter study, neutrophils were activated with the bacterial peptide, formyl-methionine-leucine-phenylalanine (fMLP), a known neutrophil agonist. Whether or not such exosome-mediated pathological responses occur following nanomaterial exposure merits close attention; several studies have shown that CNTs cause pulmonary inflammation as well as airway remodeling^38^. In a seminal study, Zhu et al.^39^ reported that exosomes were generated in significant numbers in the lungs of mice exposed to iron oxide nanoparticles, and noted that the exosomes were quickly transferred to the systemic circulation, thereby conveying immune activation in extrapulmonary organs. The authors inferred that the exosomes were of macrophage origin, although further studies are warranted to discern the source of nanoparticle-induced exosomes and whether neutrophils are also involved. Studies have shown that different metal or metal oxide nanoparticles may elicit varying cellular patterns of inflammation and one cannot a priori assume that macrophages are the only cell type at play^40^. In another recent study, ZnO nanoparticles were found to trigger neutrophilic inflammation in rats and numerous microRNAs were shown to be selectively up- or downregulated in serum exosomes from ZnO-exposed animals when compared with controls^41^. Using single-particle inductively coupled plasma-mass spectrometry and other techniques, Logozzi et al.^42^ reported that primary human macrophages are capable of endocytosis of gold nanoparticles (20 nm) with subsequent discharge of the nanoparticles via exosomes. Further studies are required to determine whether this is a general phenomenon and to what extent the exosomal content of nanoparticles correlates with the delivered dose. Nevertheless, it is conceivable that exosomes could be exploited as biomarkers of exposure to nanoparticles^42^.

## Neutrophil traps: a necessary nuisance?

Brinkmann et al.^43^ reported 15 years ago that neutrophils kill pathogens extracellularly by releasing so-called neutrophil extracellular traps or NETs. NETs are comprised a backbone of nuclear chromatin decorated with antimicrobial proteins such as MPO and NE. In addition to the classical or most commonly studied form of NADPH oxidase-dependent NETs, which contain nuclear chromatin, some studies have shown that neutrophils under certain conditions release NETs comprising mitochondrial DNA^44,45^.

NET formation is frequently viewed as a specialized form of neutrophil cell death that is distinct from apoptosis and necrosis^46^, and this cell death has been dubbed NETosis. This has led to some confusion in the literature, as the term NETosis is commonly equated with NET formation, and to further compound the situation, some authors refer to “vital NETosis”^47,48^. Indeed, as the terms suicidal and vital NETosis are controversial, it is advisable to simply refer to neutrophil formation of NETs with or without attendant cell death. On the other hand, it is well established that the stimulation of neutrophils with phorbol 12-myristate 13-acetate (PMA) leads to a caspase-independent, non-apoptotic form of cell death^49,50^. Recent studies suggest some commonalities between NET formation and other forms of programmed cell death (Box 1). Hence, gasdermin D, a pore-forming protein and a key executor of pyroptosis, is required for NET formation in neutrophils stimulated with PMA^51,52^. Furthermore, anti-neutrophil cytoplasmic antibodies trigger NETs via receptor-interacting protein kinase 1/3 and MLKL, key factors in necroptosis^53^, although it is important to note in this context that different stimuli may trigger different pathways of NET formation^54,55^.

NETs are thought to play a role during infection by allowing neutrophils to capture and kill pathogens extracellularly^43,56^. However, mounting evidence suggests that uncontrolled or excessive production of NETs, or defective degradation or removal of NETs, is related to the exacerbation of inflammation and the development of several diseases^57^. Hakkim et al.^58^ reported that impairment of DNaseI-mediated NET degradation is associated with systemic lupus erythematosus. Excessive formation of NETs, on the other hand, could clog blood vessels and provide a scaffold for thrombus formation^59^, whereas a recent study has shown that both DNase1 and DNase1-like 3 are capable of degrading NETs in circulation^60^. We found that NETs are handled differently by macrophages and dendritic cells with LL-37-dependent uptake followed by intracellular degradation in the former case, and extracellular, DNase1L3-mediated degradation of NETs in the latter case (Lazzaretto et al., manuscript in preparation). NETs were shown to prime T cells and reduce the activation threshold to specific antigens^61^. TLR9, an intracellular sensor that functions to alert the immune system of viral and bacterial infections by binding to DNA, was not involved. Nevertheless, this suggests that NETs may serve as a link between the innate and adaptive immune system. Furthermore, and in support of this notion, a recent study showed that deposition of cell-free DNA through neutrophil formation and ejection of NETs occurs at the site of immunization and drives the activity of aluminum adjuvant (alum), thereby enhancing adjuvant-induced adaptive immune responses^62^.

Can nanomaterials trigger NETs? Early work suggested that rod-shaped gold nanoparticles, as well as cationic lipid nanoparticles, are capable of triggering NETs, but compelling evidence was not presented as it is difficult to distinguish between neutrophil cell death with (passive) release of intracellular contents vs. the production of NETs^63,64^. Naturally, endotoxin contamination also needs to be excluded. More recently, agglomerates of endotoxin-free superparamagnetic iron oxide nanoparticles (SPIONs) were shown to elicit NETs, albeit at a very high concentration (200 µg/mL)^65^. Importantly, stabilization of the SPIONs with human serum albumin prevented NET formation. The authors also found that agglomerates of SPIONs triggered NET formation in vivo in an animal model, and that the particles were “glued” together by the NETs, and they suggested that such SPION-NET co-aggregates might occlude blood vessels^65^. The study highlights the need for careful particle design and passivation strategies to make nanoparticles safe for intravenous use. Muñoz et al.^66^ reported that nanodiamonds (10 nm) cause plasma membrane damage and signs of lysosomal instability in neutrophils, and found that these nanoparticles triggered the formation of NETs at high concentrations of nanoparticles (200 µg/mL). In contrast, larger particles (100–1000 nm) were relatively inert. The smaller particles also triggered inflammation following subcutaneous injection of a high dose (1 mg) into the foot pads of mice, but the swelling was resolved after 1–2 weeks^66^. The relevance of these findings is difficult to judge due to the high doses that were applied. It is worth noting that nanodiamonds were recently shown to be well-tolerated in sub-acute and chronic duration studies in rodents and non-human primates following intravenous injections^67^.

**Fig. 1:**
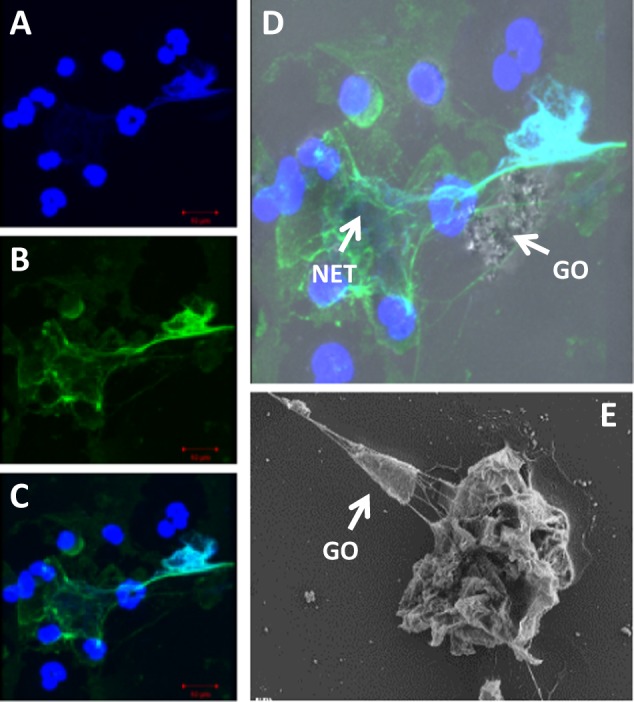
Neutrophils capture graphene oxide sheets in extracellular traps. **a**–**c** Confocal images of neutrophils incubated in the presence of large GO sheets (12.5 μg/mL). Cells were stained with antibodies to neutrophil elastase (NE) followed by a secondary FITC-labeled antibody (green) and counterstained with DAPI (blue) for visualization of cell nuclei. **d** Light and fluorescence microscopy images superimposed to show the presence of GO. **e** Scanning electron microscopy (SEM) image of neutrophils exposed to GO (12.5 μg/mL). The arrow points to a large GO sheet that has been “captured” in a network of chromatin fibers (i.e., NETs). Reproduced from Mukherjee et al.^68^, with permission from Elsevier

We recently provided first evidence that graphene oxide (GO) can trigger NETs in primary human neutrophils, indicating that the immune system can “sense” even a two-dimensional material (Fig. 1). Hence, we could show that low doses of micrometer-sized GO sheets triggered NETs in primary human neutrophils more effectively than small GO with nanosized lateral dimensions^68^. Both materials were produced under sterile conditions and were proven to be endotoxin-free. Interestingly, the large GO sheets initiated the oxidation of cholesterol in the plasma membrane of neutrophils as evidenced by time-of-flight secondary ion mass spectrometry (ToF-SIMS) furthermore, the release of NETs was reduced by Trolox, a potent lipid antioxidant^68^. Neumann et al.^69^ previously reported that methyl-β-cyclodextrin (MβCD), a cholesterol-depleting agent, triggered the formation of NETs in a manner that was independent of the NADPH oxidase (i.e., insensitive to pharmacological inhibition using diphenylene iodonium [DPI]). To study the signaling pathway underlying the formation of NETs in GO-exposed cells, we explored the effect of DPI on NET formation in neutrophils exposed to GO, PMA, or MβCD. DPI was found to block PMA-induced production of NETs, as expected, and blocked the production of NETs in neutrophils exposed to the small GO sheets^68^. However, NET formation in MβCD-treated cells and in cells incubated with large GO was unaffected by DPI, indicating that NADPH oxidase activation is not required. Interestingly, the mitochondria-targeted antioxidant MitoTEMPO significantly reduced the production of NETs by GO. Mitochondrial reactive oxygen species (ROS) are also required for calcium ionophore-induced NETs^70^. In a companion paper, we showed that small and large GO are degraded in an MPO-dependent manner in NETs purified from activated neutrophils^71^. Neutrophils can also enzymatically digest CNTs (discussed below). Thus, it appears that neutrophils are capable of handling at least some carbon-based nanomaterials as pathogens, leading to the destruction of the offending agents^72^.

## Neutrophil degradation of nanomaterials

**Fig. 2:**
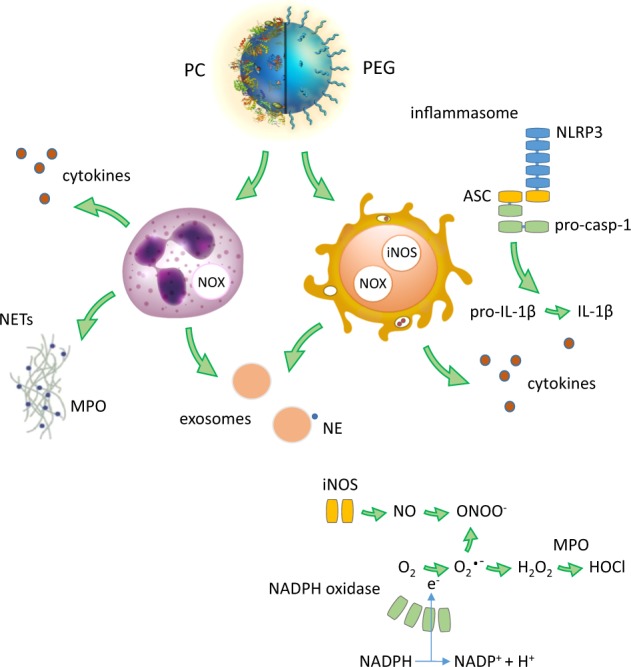
Nano-bio interactions: from coronation to degradation. This schematic diagram depicts nanoparticles with or without a protein corona (PC) and/or a polymer coating (i.e., poly(ethylene glycol) or PEG) interacting with neutrophils (left) vs. macrophages (right). Neutrophils release neutrophil extracellular traps (NETs) consisting of nuclear chromatin decorated with granule proteins such as myeloperoxidase (MPO), and recent studies have shown that carbon nanotubes (CNTs) and graphene oxide (GO) are captured and digested in NETs in an MPO-dependent manner^71,78^. The NADPH oxidase (commonly abbreviated as NOX) is a multiprotein complex expressed in phagocytes that catalyzes the generation of superoxide. Superoxide, in turn, dismutates to form hydrogen peroxide and MPO catalyzes the formation of hypochlorous acid, a freely diffusible oxidant that is microbicidal and also is responsible for the degradation of carbon-based nanomaterials^124^. In addition, superoxide and nitric oxide, produced by inducible nitric oxide synthase (iNOS), react to form peroxynitrite, which was shown to digest nanomaterials in macrophages^125^. Macrophages emit pro-inflammatory IL-1β through an inflammasome-dependent mechanism (a cytosolic protein complex shown outside the cell for clarity). Neutrophils and macrophages release exosomes, thus providing a further means of cell-to-cell communication and propagation of inflammation. Recent work has shown that neutrophil-derived exosomes express neutrophil elastase (NE) on the surface; these exosomes degrade extracellular matrix more readily when compared with free NE^37^

The membrane-bound NADPH oxidase generates ROS that are instrumental for the killing of ingested pathogens. Degranulation with the release of MPO is also an important feature of the microbicidal actions of neutrophils^73^. In addition to its role in antimicrobial defense, MPO is also reported to be involved in the degradation of CNTs and the clearance of CNTs from the lungs was markedly less efficient in MPO-deficient mice when compared with wild-type mice^74^. Neutrophil-mediated destruction of CNTs was first described by Kagan et al.^75^. Subsequently, MPO-dependent degradation of PEGylated CNTs was reported and this was suggested to occur in a two-step process whereby the polymers were first removed from the CNTs by NE followed by the degradation of the CNTs themselves by MPO^76^. Eosinophils can also digest CNTs^77^. Furthermore, CNTs and GO were shown to undergo acellular degradation in NETs purified from activated neutrophils^71,78^ (Fig. 2). In a recent study, GO functionalized with fMLP was shown to stimulate neutrophil degranulation leading to degradation (Martin et al., manuscript in preparation). Even graphene can undergo neutrophil-mediated degradation, although the process is considerably slower when compared with GO^79^. The latter studies were conducted ex vivo using human neutrophils. Notably, a recent in vivo study has shown that GO (20 mg/kg) administered subcutaneously elicits an inflammatory reaction in mice in response to implantation consistent with a foreign body reaction^80^. The latter study did not evaluate biodegradation of the implanted materials. Girish et al.^81^, on the other hand, explored biodegradation after intravenously injecting graphene (20 mg/kg) into mice by using a Raman confocal imaging approach. The authors noted graphene engulfment by tissue-bound macrophages and found that degradation was prominent after 90 days. Tuning the properties of graphene-based materials to achieve optimal performance while maintaining an acceptable degree of biocompatibility and biodegradability remains an important challenge in the field^82^.

## Inflammasomes: double-edged swords

How do nanomaterials and other exogenous substances trigger inflammation? Numerous studies have shown that the inflammasome, originally described by Tschopp and colleagues^83^, is a key signaling hub that regulates innate immunity and inflammation^84^. The inflammasomes are multiprotein complexes that are activated in response to diverse pathogen- and host-derived danger signals leading to the activation of caspase-1 with processing of cytosolic pro-IL-1β and secretion of pro-inflammatory IL-1β^85^. NLRP3, in particular, responds to a diverse array of different stimuli including crystalline and particulate matter such as uric acid crystals, silica, asbestos, and alum, as well as to pathogens^86–89^. NLRP3 is also a sensor of various nanomaterials^90^. Indeed, not only long and rigid CNTs but also ultrathin GO sheets and spherical carbon particles are able to trigger NRLP3-dependent IL-1β secretion in human macrophages^91–93^ (Fig. 2). However, less is known regarding inflammasome activation in neutrophils. Neutrophils are capable of activating the NLRC4 inflammasome in response to bacterial challenge, but this occurs without the induction of pyroptosis^94^. On the other hand, NE cleaves gasdermin D in neutrophils^95^, thus providing an alternative route to pyroptosis in these cells. Indeed, it should be noted that the production of IL-1β is not exclusively dependent on caspase-1^96^. Thus, neutrophil-derived serine proteases are also involved in the processing of IL-1 family cytokines, and serine proteases and/or caspases may be involved in neutrophils depending upon the stimulus^97^.

How big is a speck? ASC (apoptosis-associated speck-like protein containing a CARD) is a CARD (caspase recruitment domain) carrying protein of 22 kDa that is involved in the recruitment of pro-caspase-1 to the inflammasome. ASC was described 20 years ago as a protein that could be visualized as a small spot or speck in the cytosol of apoptotic cells^98^. Intriguingly, although inflammasome activation was originally believed to take place in the cytosol of cells, subsequent studies have shown that cells may transmit inflammation in a prion-like manner via extracellular ASC. These micrometer-sized clumps of ASC proteins continued to stimulate caspase-1 activation extracellularly and stimulated further inflammasome activation in neighboring macrophages that had ingested the ASC oligomers^99,100^. Extracellular ASC was found in tissues of patients with inflammatory diseases and autoantibodies to ASC developed in some patients with autoimmune pathologies. Thus, as pointed out previously, danger signals come in many shapes and sizes^101^. It should therefore not come as a surprise that the immune system is capable of responding to synthetic (nano)particles.

## Decoding danger at the nanoscale

**Fig. 3:**
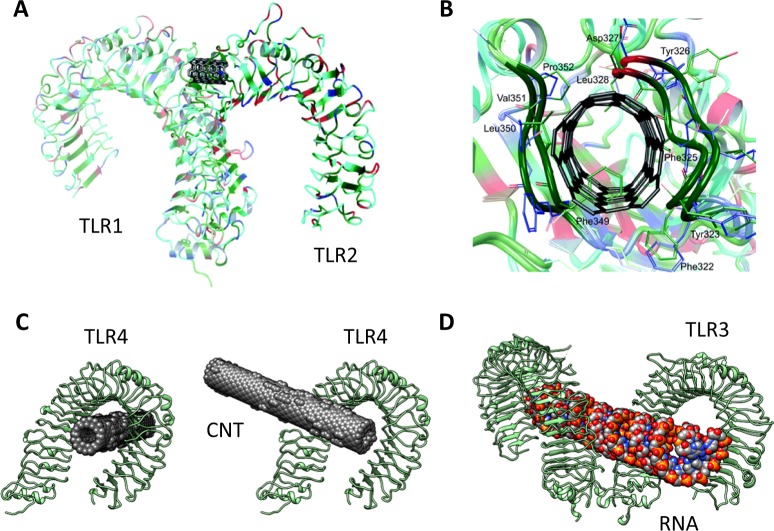
Macrophage sensing of single-walled carbon nanotubes as pathogens. Computational modeling of a SWCNT bound to the extracellular domains of TLR1/TLR2 (**a**). The nanotube is surrounded by amino acids of mostly hydrophobic nature giving rise to strong van der Waals interactions (**b**). The results shown in **c** depict one of the best binding poses obtained by molecular docking of carboxylated SWCNTs with TLR4^117^. Interestingly, the highest scoring binding mode of SWCNT and TLR4 shared several similarities with the experimentally resolved structure of TLR3 in complex with double-stranded RNA (**d**)^126^. These findings suggest that TLR4 homodimers may engage with SWCNTs through a tweezer-like mechanism. It is noted that these modeling results were derived in the absence of a protein corona in order to elucidate direct binding to TLRs. The efficiency of protein adsorption is well-known to be proportional to the diameter of the nanotubes^127^. Therefore, the small diameter of these SWCNTs may limit protein adsorption, thus leaving a sufficient surface for the direct interaction with TLRs^117^. Panel **a** and **b** are from Turabekova et al.^118^ with permission from The Royal Society of Chemistry. Results shown in panel **c** are from ref. ^117^ while results in panel d were generated based on ref. ^126^

Cells of the immune system are equipped with PRRs that monitor the extracellular or intracellular environment for signs of infection or “danger”. Toll-like receptors (TLRs) are present both on cell surfaces and in endosomal compartments, whereas retinoid acid-inducible gene-I-like receptors and nucleotide-binding and oligomerization domain-like receptors (NLRs) are present in the cytosol^102^. Furthermore, soluble scavenging receptors have been described^103,104^. PRRs presumably evolved to discriminate between foreign intruders and “self,” but they also recognize DAMPs released from stressed or damaged cells^105^. It has been estimated that a single cell may express as many as 50 distinct PRRs, thus testifying to the importance of sensing “danger”^106^. Nanomaterials—with or without a corona of proteins or other biomolecules—may be considered as a particular case of danger signals that are able to trigger sterile inflammatory responses. Indeed, we have previously postulated that engineered nanomaterials may present nanomaterial-associated molecular patterns or NAMPs to cells of the immune system^107^. Thus, in analogy with microorganisms (bacteria, viruses) displaying PAMPs and damaged or stressed cells releasing DAMPs, we postulated that engineered nanomaterials coated with a corona of biomolecules may act as nanoparticle-associated molecular patterns or NAMPs^107^. The notion of nanomaterial-associated molecular patterns has captured the attention of several other authors^108–111^. The fact that nanoparticles with an adsorbed corona of proteins may display epitopes that are sensed by the immune system is of considerable interest, as this may point toward a systematic understanding of nano-bio interactions^112^. It may also be worthwhile to explore whether nanoparticles per se present molecular patterns that are decoded by the immune system. Indeed, emerging studies suggest that some nanoparticles could act as protein mimics capable of engaging with intra- or extracellular receptors^113^, and the combination of experimental and theoretical studies promises to shed light on this exciting topic^114,115^. Using a proteomics approach, He et al.^116^ found that carbon-based nanomaterials (i.e., single-walled carbon nanohorns, SWCNTs, and MWCNTs) bound to glycoprotein nonmetastatic melanoma protein B (GPNMB, also known as osteoactivin) in macrophages. The authors suggested that GPNMB serves as an intracellular PRR for these nanomaterials. We have recently demonstrated, by using a transcriptomics approach, that SWCNTs prompted the upregulation and secretion of chemokines in primary human macrophages, and we provided evidence for direct binding of CNTs to TLR2/4^117^. The nanomaterials used were endotoxin-free. Taken together, these results give credence to the idea that nanomaterials may act as NAMPs^107^. Interestingly, computational studies predicted that the binding of carbon-based nanostructures to proteins is guided mainly by hydrophobic interactions^114^. More specifically, we and others have analyzed the association of CNTs and other carbon nanostructures to TLRs^117,118^. Turabekova et al.^118^ predicted that the hydrophobic pockets of some TLRs might be capable of binding pristine SWCNTs and C_60_ fullerenes (Fig. 3). Furthermore, we suggested that ion-pair interactions with positively charged residues might strengthen the binding of carboxylated CNTs to TLRs^117^. It will certainly be important to study whether engineered nanomaterials also engage with neutrophil PRRs.

It is worth noting that nanoparticles can also be exploited for the removal of DAMPs such as cell-free DNA that is expelled from dying cells to ameliorate inflammatory diseases initiated by the inappropriate activation of TLR signaling. Hence, Liang et al.^119^ prepared cationic nanoparticles composed of the block copolymer of PLGA and poly(2-(diethylamino)ethyl methacrylate), and found that these particles had a high DNA-binding capacity. Furthermore, when injected intravenously the cationic nanoparticles could alleviate symptoms in animal models of arthritis. These results, along with previous work by other investigators, suggest that cationic nanoparticles may act as nucleic acid scavengers^120,121^. Further studies are needed to formally address whether the acquisition of a protein corona on scavenger particles navigating the blood stream would interfere with or promote nucleic acid binding.

## Concluding remarks

Neutrophils are key effector cells of the innate arm of the immune system and play important roles in host defense against pathogens, and yet, paradoxically, they are also involved in numerous pathological conditions characterized by chronic inflammation. Studies in recent years have shown that nanomaterials can modulate and activate neutrophils and other immune cells. Moreover, activated neutrophils may capture and digest certain carbon-based nanomaterials. Neutrophils also play a role in particle clearance in the systemic circulation (at least in mice). Understanding the interactions between nanomaterials and neutrophils is important for the development of safe and effective nanomaterials for biomedical applications.

The nanotoxicology literature is replete with publications on the negative impact of nanomaterials, often referencing the pro-inflammatory effects of the materials, even when studies are performed in cell culture where coordinated immune reactions cannot occur. However, it is important to note that inflammation as such is not a detrimental response. Therefore, one should not seek to prevent (acute) inflammation at every cost. Instead, careful design of nanomaterials is required in order to avoid chronic, adverse reactions. Furthermore, nanomaterials may be exploited to harness immune responses to ameliorate chronic inflammation and/or autoimmune diseases, and leverage immune responses toward cancer cells^122,123^.
